# Elevated CO_*2*_ Reduced Floret Death in Wheat Under Warmer Average Temperatures and Terminal Drought

**DOI:** 10.3389/fpls.2015.01010

**Published:** 2015-11-20

**Authors:** Eduardo Dias de Oliveira, Jairo A. Palta, Helen Bramley, Katia Stefanova, Kadambot H. M. Siddique

**Affiliations:** ^1^CSIRO Agriculture FlagshipWembley, WA, Australia; ^2^School of Plant Biology, Faculty of Natural and Agricultural Sciences, The University of Western AustraliaCrawley, WA, Australia; ^3^The UWA Institute of Agriculture, The University of Western AustraliaCrawley, WA, Australia; ^4^Plant Breeding Institute, Faculty of Agriculture and Environment, The University of SydneyNarrabri, NSW, Australia

**Keywords:** *Triticum aestivum*, restricted tillering, free -tillering, climate change, yield, compensation, florets

## Abstract

Elevated CO_2_ often increases grain yield in wheat by enhancing grain number per ear, which can result from an increase in the potential number of florets or a reduction in the death of developed florets. The hypotheses that elevated CO_2_ reduces floret death rather than increases floret development, and that grain size in a genotype with more grains per unit area is limited by the rate of grain filling, were tested in a pair of sister lines contrasting in tillering capacity (restricted- vs. free-tillering). The hypotheses were tested under elevated CO_2_, combined with +3°C above ambient temperature and terminal drought, using specialized field tunnel houses. Elevated CO_2_ increased net leaf photosynthetic rates and likely the availability of carbon assimilates, which significantly reduced the rates of floret death and increased the potential number of grains at anthesis in both sister lines by an average of 42%. The restricted-tillering line had faster grain-filling rates than the free-tillering line because the free-tillering line had more grains to fill. Furthermore, grain-filling rates were faster under elevated CO_2_ and +3°C above ambient. Terminal drought reduced grain yield in both lines by 19%. Elevated CO_2_ alone increased the potential number of grains, but a trade-off in yield components limited grain yield in the free-tillering line. This emphasizes the need for breeding cultivars with a greater potential number of florets, since this was not affected by the predicted future climate variables.

## Introduction

Atmospheric CO_2_, ambient temperature and rainfall are environmental variables predicted to change in Mediterranean-type environment in the in the 21st century with climate change (Ragab and Prudhomme, 2002; Asseng et al., 2004). Rainfall in particular, is predicted to decrease when wheat crops enter the reproductive growth (terminal drought), reducing grain yield (Ludwig and Asseng, 2006; Turner et al., 2011). In a previous study, grain yield in a free-tillering wheat line, with the potential to increase sink capacity, did not differ from its isogenic restricted-tillering line, with lower sink capacity. This was because the free-tillering line increased grain number per unit area due to elevated CO_2_, but had smaller grains (Dias de Oliveira et al., 2015), presumably due to the negative correlation between grain number per unit area and grain size (Grafius, 1972). Increasing grain number in wheat is considered critical for improving grain yield (Fischer and Aguilar, 1976; Fischer, 1985, 2011), but it is also likely that grain yield improvement may be limited by grain size (Grafius, 1972; Rajaram and Ginkel, 1996; Acreche and Slafer, 2006). Understanding the mechanisms determining grain number per unit area and grain size is, therefore, important in simultaneously increasing both yield components (Reynolds et al., 2009; Fischer, 2011; Ferrante et al., 2013b).

Grain number per unit area in wheat is determined by ear number per unit area and number of grains per ear. Final grain number per ear is highly affected by the availability of assimilates before anthesis, since modern cultivars set more than 90% of the potential grains at anthesis (Siddique et al., 1989). Reduction of assimilates before anthesis due to heat and water stress, and nitrogen deficiency reduces potential grain number at anthesis (Calderini et al., 2001; Ferrante et al., 2013a). In contrast, increased availability and distribution of carbon assimilates increases yield potential (Slafer, 2003; Reynolds et al., 2009). Elevated CO_2_ increases the assimilation of carbon in wheat (Habash et al., 1995) and presumably the availability of carbon assimilates for floret development (Marc and Gifford, 1984), potential grain number, and hence, more grains per unit area. Elevated CO_2_ often increases grain number per ear, possibly due to increased floret production and/or a reduction in floret death (Musgrave and Strain, 1988; Manderscheid and Weigel, 1997; Dias de Oliveira et al., 2013, 2015). However, in some cases no CO_2_ effect on grain number per ear have been reported (Högy et al., 2009, 2013), presumably reflecting genotypic variability to treatment. The amount of carbon assimilates needed to produce florets is almost insignificant compared with the amount needed to sustain their growth and development (Kirby, 1988; Ferrante et al., 2013a). This implies that increasing the availability of carbon assimilates should benefit floret survival rather than floret production.

This study aimed to determine the mechanisms by which grain number increased in the main stem of wheat when grown under elevated CO_2_, warmer average temperatures and terminal drought, which are the predicted conditions of future climates in the wheat-growing regions of Australia. It also aimed to understand the mechanism responsible for reducing grain weight (yield component compensation) in two sister lines of wheat contrasting in tillering capacity and hence, in grain number per unit area. Two hypotheses were tested: (i) elevated CO_2_ increases grain number per ear by reducing the rate of floret mortality due to the availability of more carbon assimilates to support competent florets, and not by increasing the maximum number of florets produced; (ii) the rate of grain filling in a free-tillering line is slower than in a restricted-tillering line, resulting in smaller grains in the line with more grain per unit area. To test these hypotheses, two sister lines of wheat contrasting in tillering were grown under elevated CO_2_ (700 μL L^-1^), +3°C above ambient temperature and terminal drought conditions in poly tunnels, and the dynamics of tillering, floret, and grain development was monitored.

## Materials and Methods

### Location

A field experiment was conducted between May and November 2012 at The University of Western Australia’s (UWA), field Research Station at Shenton Park (31° 57′S, 115° 45′E), Western Australia. The site is located in an area with a long-term average rainfall (80 years) of 710 mm for the May–November growing season. The soil is a free-draining infertile Spearwood clayey sand (McArthur and Bettenay, 1974) consisting of brown, fine sandy clay with less than 1% organic matter. The pH, measured in a 1:5 suspension of soil in 0.01 M CaCl_2_, was 5.1–6.8 in the surface 0–10 cm and 4.1–4.5 in lower layers of the soil profile.

### Plant Material

A pair of sister lines of wheat (*Triticum aestivum* L.), representing contrasting genotypes for tillering capacity (free tillering and restricted tillering), were used in this study. The lines 7750N (free tillering) and 7750PF (restricted tillering), selected by Dr G Rebetzke at CSIRO Plant Industry, were F5-derived, sister lines for restricted tillering in a Lang genetic background (Wyalkatchem//3^∗^Silverstar/971/3/Lang). The pair of sister lines were grown in the field under four poly tunnels in a completely randomized design with elevated CO_2_, high temperature and terminal drought in all combinations in a split-plot design within each poly tunnel. One day before seeding, the 20 plots (1.0 m × 0.75 m) in each poly tunnel were cultivated by hand to a depth of 6 cm using a wide rake. Plants of each sister line (10 plots per line) were sown by hand to attain a density of 150 plants m^-2^ on 21 May 2012. The equivalent of 60 kg N ha^-1^ as urea, 75 kg P ha^-1^ as amended superphosphate (with Cu, Zn, Mo, S) and 55 kg K ha^-1^ as KCl was buried with the seed. A top-dressing application of 33 kg N, 38 kg P, and 28 kg K ha^-1^ was made when plants were at the 3–4 leaf stage (Z13–Z14; Zadoks scale of growth; Zadoks et al., 1974). An additional application of N as urea was made at the end of stem elongation (Z39) at a rate of 50 kg N ha^-1^.

### Natural Light-Controlled Temperature, CO_2_ and Irrigation Field Facilities (NL-CTCI)

Details of the NL-CTCI facilities were described in Dias de Oliveira et al. (2013). Briefly, the NL-CTCI consist of four poly tunnels, each of 10 m long × 2.5 m wide × 2.75 m tall, supported by a steel frame covered with a double sheet of F-clean greenhouse film (200 mm; AGC Chemicals Americas, Inc. Exton, PA, USA). Air flow through each tunnel was provided by a CPD 0454 FHP multi-speed fan (Fantech, Melbourne, VIC, Australia), which moved the outside air through a cardboard radiator window mounted in the opposite wall of the tunnel. The fan speed was varied to maintain a set temperature inside the tunnel, monitored with a Techni-temp Resistant Thermometer Detector (Technitemp, Kewdale, WA, Australia; model TWA 27708) at the end of the tunnel. Certified CO_2_ gas was pulsed from a gas vessel (BOC Special Gasses, Chatswood, NSW, Australia) through a finely perforated plastic hose connected to a solenoid valve in the inlet stream of each tunnel. The rate of solenoid valve pulse was determined by measuring the CO_2_ level in the outlet stream of the tunnel, using a GAS-CO_2_-002-K infrared gas analyzer (Gas Alarm System, Sydney, NSW, Australia). CO_2_ concentration inside the tunnels was maintained at 700 μL L^-1^ with no discernible gradients of CO_2_ or temperature along the tunnels at any time. When a tunnel was set to ambient CO_2_ and temperature, the fan moved air though the tunnel with CO_2_ concentrations from outside of ∼390 mL L^-1^ and fan speed was determined by continuously monitoring air temperature outside the tunnel. A system of drippers disposed in rows irrigated the plots within each tunnel, with the capacity to release 2 L m^-2^ h^-1^.

### Experimental Design and Treatment Structure

A randomized complete block design was used to address the objectives of the study and reflect the treatment structure.

Each poly tunnel served as a combination of CO_2_ and temperature regime, as follows:

(1) Ambient CO_2_ (390–400 μL L^-1^) + ambient temperature (ACO_2_ + AT)
(2) Ambient CO_2_ (390–400 μL L^-1^) + 3°C above ambient temperature (ACO_2_ + HT)
(3) Elevated CO_2_ (700 μL L^-1^) + ambient temperature (ECO_2_ + AT)
(4) Elevated CO_2_ (700 μL L^-1^) + 3°C above ambient temperature (ECO_2_ + HT).

The poly tunnel comprised of 20 plots arranged in a rectangular array of 2 columns by 10 rows. Each column within a tunnel was randomly allocated to a different water regime from anthesis (watered or water stressed). The 10 plots within each column were completely randomly assigned to one of the two sister lines, producing five replicates per sister line for each of the treatment combinations. Plots inside each tunnel were irrigated every 2–3 days by drip irrigation to maintain soil water content close to field capacity until 50% anthesis. At anthesis irrigation was withheld from half of the plots in each poly tunnel house (10 plots; 5 per sister line) to induce terminal drought. The other half of the plots (10 plots; 5 per sister line) was maintained under irrigation until physiological maturity (well-watered). Well-watered and droughted plots were separated by a vertical plastic barrier buried to 1.2 m in the soil profile to prevent lateral movement of water from the well-watered to the droughted plots. The well-watered plots received approximately 450 mm m^-2^ of water during the whole season (similar to the 1995–2014 rainfall average for this location).

### Sampling and Measurements

The dates of the developmental stages (phenostages) for anthesis and physiological maturity (flag leaves had turned yellow; Hanft and Wych, 1982) were recorded for each plot in each poly tunnel. Daily observations were made and the phenostage noted when 50% of the plants in each plot had achieved the particular stage. Phenostages were defined using the Zadoks’ scale of cereal development (Zadoks et al., 1974). Comparisons between lines in each pair were made in days after sowing (DAS).

The measurements taken in time for each response variable allowed non-linear curve fitting modeling of the plant dynamics. The notation used in each of the curve formulae presented below denotes the response variable *y* as a function of thermal time after sowing or anthesis, the latter denoted as *x*. Thermal time is the accumulated temperature at which plant growth responds to the Zadoks’ phenostages.

Production of stems was recorded in five uniform plants per plot, averaged and treated as one replicate (*n* = 5). To identify order of emergence, the main stem and tillers were each labeled with a thin plastic ring (drinking straw) of differing color. Tillering dynamics for both lines were modeled by a quadratic-by-quadratic curve, which accounted for 90.6% of the variation.

$$y\,\;=\;\frac{a\,\;\;+\;\;(b\,\;\;+\;\;cx)}{\left(1\,\;\;+\;\;dx\,\;\;+\;\;ex^{2}\right)},\;\;\;\;\;\;\;\;\;\;\;\;\;\;\;\;\;\;\;\;\;\;\;\;\;\;\;\;\;\;\;\;\;\;\;\;(1)$$

The number of florets in spikelets 8 and 10, the mid-ear for most wheat genotypes (Bancal, 2009), were counted every 4 days from the beginning of stem elongation (Z3.1). This stage coincided with the beginning of floret development and terminal spikelet formation. On each occasion, ears from three main stems were sampled from each plot (averaged and treated as one replicate, *n* = 5) in each poly tunnel, dissected under a stereoscopic microscope and the number of florets counted. Ears were then oven-dried for 48 h at 70°C and weighed. The timing of floret primordia development was fitted using equation (1), which accounted for 75% of the variation from the beginning of stem elongation (Z3.1) until anthesis. The development of ears followed asymmetric asymptote growth curves, which accounted for 89.0% of the variation, were best fitted by Gompertz sigmoid function

$$y\,\;\;=\;\;a\,\;\;+\;\;\frac{c}{1\,\;\;+\;\;e^{-b(x-m)}}\,\;\;\;\;\;\;\;\;\;\;\;\;\;\;\;\;\;\;\;\;\;\;\;\;\;(2)$$

Three main stems per plot (averaged and treated as one replicate, *n* = 5) in each poly tunnel were randomly selected and tagged at the booting stage (Z4.1) for measurements of flag leaf length and ear peduncle length. Measurements were made with calipers every 2 days to determine their growth rates. Flag leaves were measured after completely unfolding, and peduncles were measured after complete ear emergence. The best fit for flag leaf expansion and peduncle extension in both sister lines was obtained using Gompertz curve (2), which accounted for 98 and 99% of the variation, respectively.

Anthesis on the main stem was recorded by daily observation in each plot in each poly tunnel and the peduncles of ears with extruded anthers from their central spikelets were tagged for grain growth measurements. A section of plastic straw cut longitudinally was used to tag the peduncles. Different colors were used for different days so that the day of anthesis could be recognized for each ear. Measurements were made on ears with the same number of days after anthesis, but data were plotted versus thermal time (°C d). Ears from three main stems per plot (averaged and treated as one replicate, *n* = 5) were sampled first at anthesis and every 4 days after anthesis (DAA), until 36 DAA. Ears were dissected and grains from spikelets 8 and 10 were sampled and weighed after oven-drying for 48 h at 70°C. Asymmetric asymptotes of grain growth curves were best fitted using equation (2), which accounted for 93% of variation. Lag phase duration of grain growth was calculated as a percentage of total grain growth time as in Loss et al. (1989).

Leaf net photosynthesis and transpiration rates of four flag leaves per plot (averaged and treated as one replicate, *n* = 5) were measured using a portable CIRAS-II open gas exchange system (PP Systems, Amesbury, MA, USA). Measurements were made from 50% anthesis at 5–6 days intervals between 10:30 and 13:30 using natural light on cloud-free days, when PPFD was above 900 mmol m^-2^ s^-1^, the level at which photosynthesis is saturated in wheat (Austin, 1990; Henson et al., 1990). The temperature and CO_2_ concentration used in gas exchange measurements were set the same as the respective poly tunnel, with the CO_2_ concentration maintained by the CO_2_ cartridge. Air-flow in the CIRAS chamber was 200 mL min^-1^ and the vapor pressure deficit was between 0.7 and 1.2 kPa. Transpiration efficiency (TE; μmol CO_2_ mmol H_2_O^-1^) was calculated as the ratio of the rate of net leaf photosynthesis divided by leaf transpiration rate.

Grain yield and yield components were measured at final harvest by sampling 25 plants per plot in each poly tunnel. Plants in each plot were assigned randomly with plants from edge rows not sampled to avoid edge effects. Plants were harvested by cutting the shoots at the surface of the soil. Plant material was separated into stems and ears, oven-dried at 70°C for at least 48 h, and then weighed. Ears were counted and threshed by hand, and grain was re-dried and weighed. Grain number was counted, and the mean individual grain weight determined for each sample. Harvest index (HI) was calculated as total grain yield divided by total aboveground biomass.

### Experimental Design and Statistical Analysis

The experimental design was a four-factorial complete randomized split-plot design, comprising two levels of CO_2_ (ambient and elevated) and two temperatures (ambient and ambient +3°C), with 10 replicates within each environment (poly tunnel) until anthesis and five replicates after anthesis. Each poly tunnel house had a split-plot combination of watering regimes (main plot; terminal drought; and well-watered) and contrasting lines (sub-plot; free–tillering and restricted-tillering lines). Grain yield and yield components were analyzed using linear mixed models techniques, accounting for the split-plot design. The results focus on the significant main and interaction effects of treatment factors and the presented tables show results only for these sources of variation (either, interactions or main effects). The repeated measures of net photosynthesis and leaf transpiration rate over thermal time (accumulation of daily temperature averages) were analyzed using generalized linear models in combination with additive linear models, where polynomial functions of time were added. In addition to the linear component of time, quadratic and, for some models, cubic components were fitted. The model with best fit was that which accounted for the biggest percentage of variance. Curve fitting was used for analysis of tillering dynamics, flag leaf, and peduncle extension, number of florets per spikelet, and ear and grain growth over thermal time. Thermal time (as the independent variable) along with a grouping factor was included in the model. The grouping factor had eight levels, combining three factors: CO_2_ concentration, temperature and watering regime. The curves were fitted separately for each genotype. Data were analyzed using GenStat 15 (VSN International Ltd, UK). Figures were made using GraphPad Prism 5.0 (GraphPad Software Inc., San Diego, CA, USA).

## Results

### Phenology

There was no difference between lines (*P* = 0.467) in time to anthesis, which averaged 83 DAS under ambient conditions. However, time to anthesis was affected by a CO_2_ × temperature interaction (*P* = 0.005), where elevated CO_2_ reduced the time to anthesis by 2 days under ambient temperature only. At physiological maturity there were no significant interactions (*P* = 0.152–0.911). Both lines reached physiological maturity by 111 DAS under ambient conditions (*P* = 0.823). The combination of elevated CO_2_ + high temperature reduced time to physiological maturity by 6 days, while terminal drought reduced time to physiological maturity by 10 days (*P* < 0.001 for all).

### Tiller Dynamics

**FIGURE 1:**
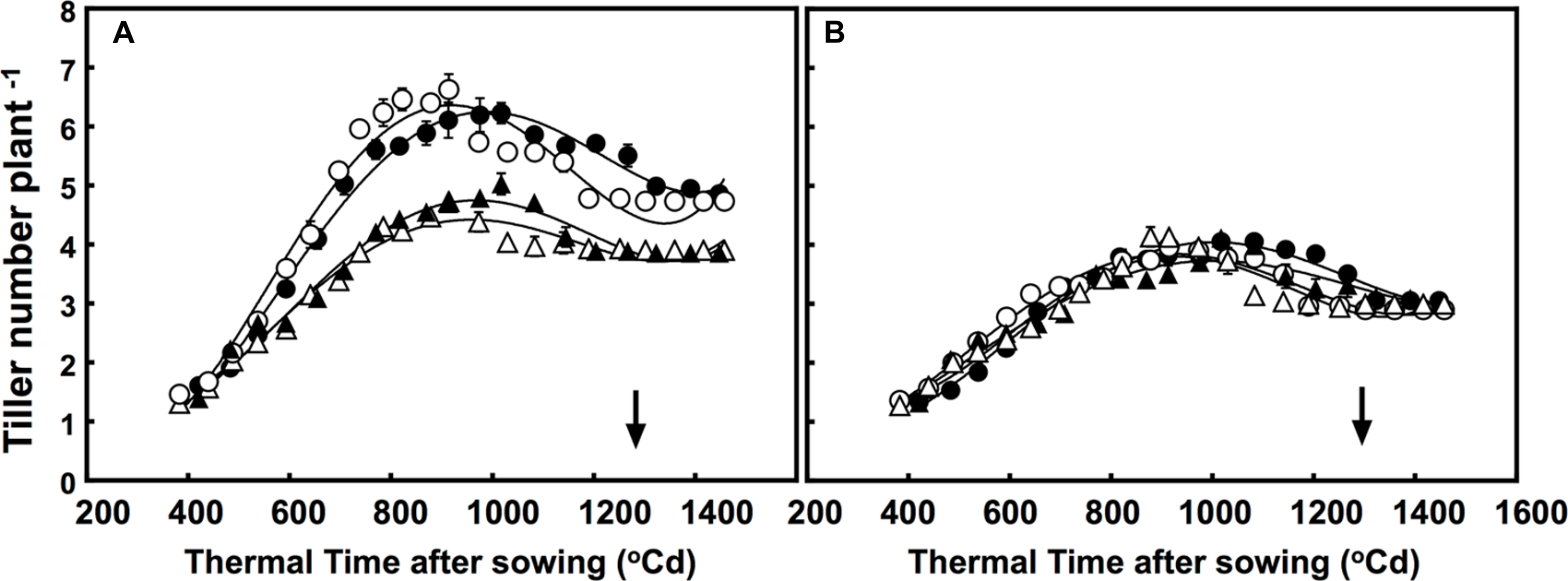
**Dynamics of tillering in the **(A)** free-tillering (FT) and **(B)** restricted-tillering (RT) sister lines**. Black triangles = ACO_2_ + HT; white triangles = ACO_2_ + AT; black circles = ECO_2_ + HT; and white circles = ECO_2_ + AT. Arrows indicate time of anthesis. Each point is mean ± SEM of 10 replicates.

Although the number of tillers were greatly different, the free-tillering line had significantly more tillers than the restricted-tillering line under all conditions. The tillering dynamic of each sister line was affected by CO_2_ × temperature (*P* = 0.001; **Figure 1**). Furthermore, elevated CO_2_ affected tillering dynamics in the free-tillering line regardless of temperature, but had no effect in the restricted-tillering line. Elevated CO_2_ increased tiller growth rate (∼0.0042% more tillers per °C d) and maximum number of tillers (35% more tillers) in the free-tillering line, but did not affect the rate of tiller death (tillers formed, but did not survive). Therefore, the free-tillering plants grown under elevated CO_2_ maintained one more tiller than plants grown under ambient CO_2_ even after flowering (*P* = 0.008; **Figure 1**). Temperatures +3°C above ambient also increased the rate of tiller development in the free-tillering line, such that when tiller number reached its maximum (approximately 1000°C d), there were on average 0.25 more tillers than under ambient temperature.

### Number of Florets

**FIGURE 2:**
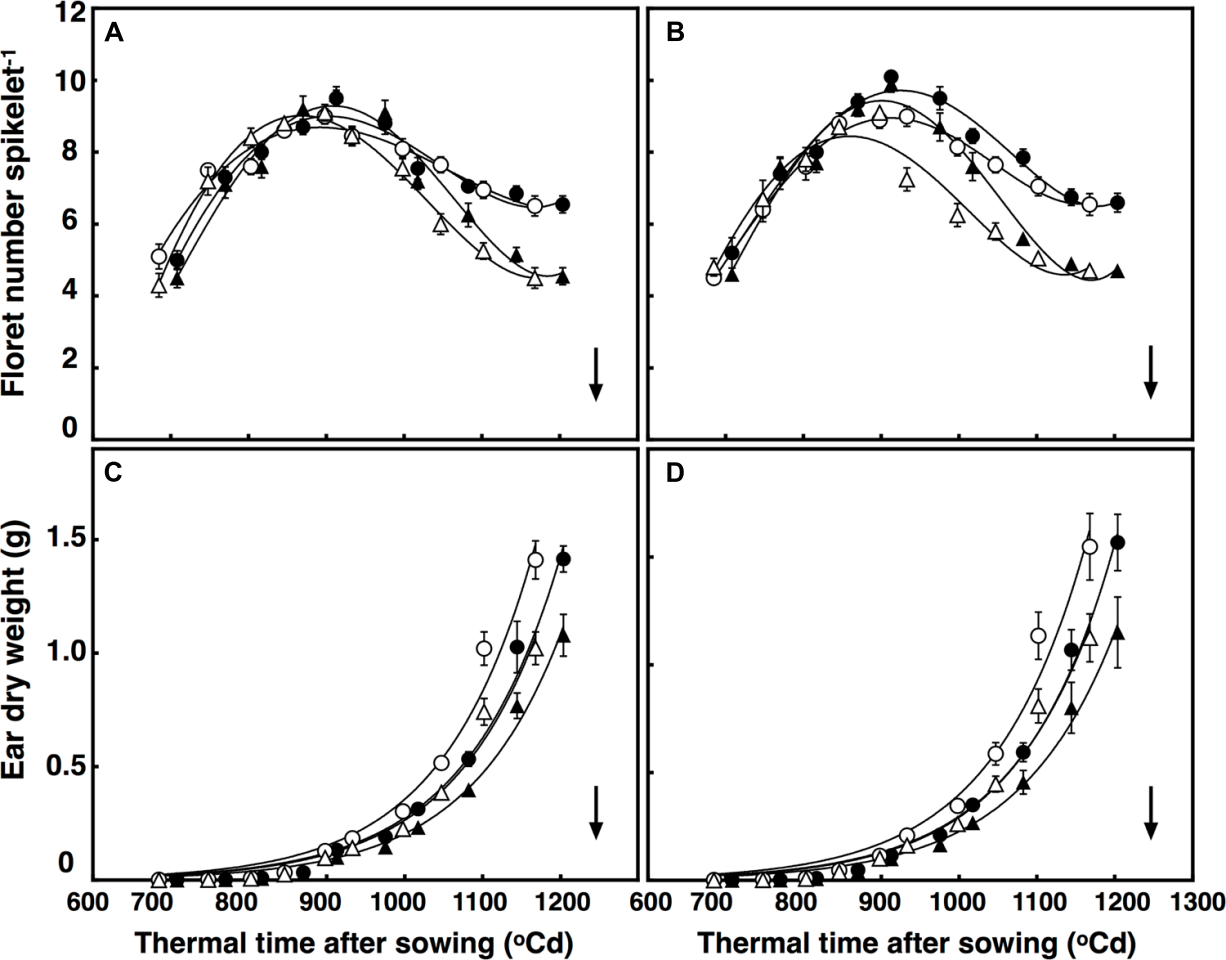
**Development of florets per spikelet **(A,B)** and ear dry weight **(C,D)** in **(A)** free-tillering and **(B)** restricted-tillering lines**. Black triangles = ACO_2_ + HT; white triangles = ACO_2_ + AT; black circles = ECO_2_ + HT; and white circles = ECO_2_ + AT. Arrows indicate time of anthesis. Each point is mean ± SEM of 10 replicates.

Elevated CO_2_ concentration reduced floret death, increasing the number of competent florets in both lines at anthesis by on average 42% (*P* < 0.001). Moreover, after reaching their maximum in all treatments at around 925°C d, different interactions affected death rates of the florets. These interactions were CO_2_ × temperature × thermal time (*P* = 0.004), and temperature × sister line (*P* = 0.015; **Figures 2A,B**). To simplify, the number of florets increased faster under +3°C above ambient in the restricted-tillering line only. In the first interaction, floret death rates under high temperature in the restricted-tillering line was higher than in the free-tillering line. In the second interaction, high temperature increased floret death under ambient CO_2_ more than under elevated CO_2_ in both lines.

### Ear Growth

Maximum growth rate of ears was synchronous with death of primordial florets in both sister lines (**Figure 2**). There was a significant interaction of thermal time × sister line on ear growth (*P* = 0.03). Ears at 800°C d (around 1 cm stage) did not differ in dry weight between the two sister lines, but the rate of ear growth was faster in the restricted- than in the free-tillering line. By anthesis, ear weight in the restricted-tillering line was 9.3% greater than in the free-tillering line (**Figures 2C,D**). A significant interaction of thermal time × CO_2_ also affected the growth of the ears (*P* < 0.001). Elevated CO_2_ increased ear weight at anthesis by 37.5% on average across both contrasting lines (**Figures 2C,D**).

### Flag Leaf and Peduncle Extension

**FIGURE 3:**
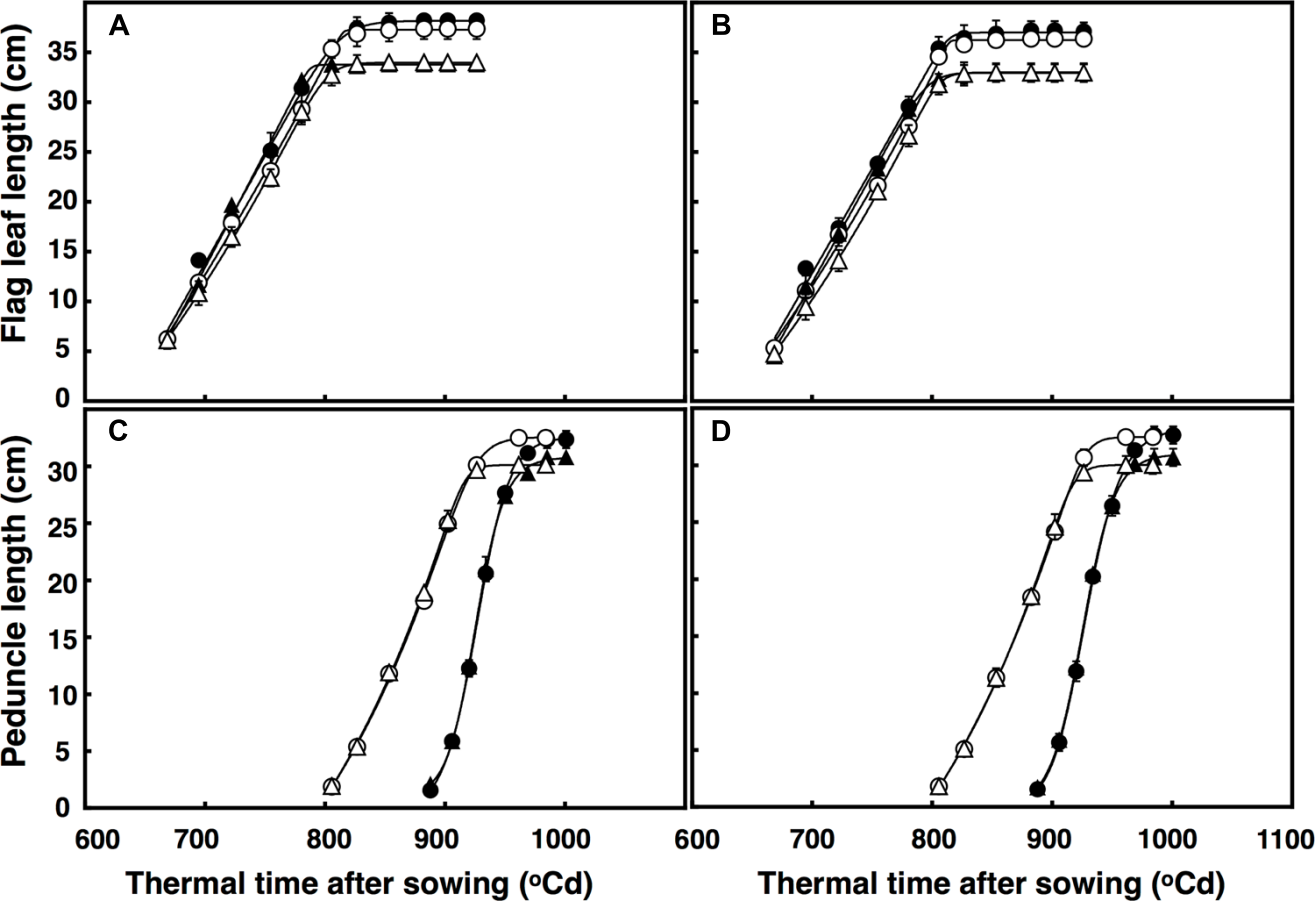
**Length of the flag leaf **(A,B)** and peduncle **(C,D)** in **(A)** free-tillering and **(B)** restricted-tillering lines**. Black triangles = ACO_2_ + HT; white triangles = ACO_2_ + AT; black circles = ECO_2_ + HT; and white circles = ECO_2_ + AT. Each point is mean ± SEM of 10 replicates.

There was a three-factor interaction of thermal time × temperature × CO_2_ (*P* = 0.014) for flag leaf expansion. The rate of flag leaf extension (∼0.35 cm per °C d) in both lines was similar regardless of CO_2_ concentration and temperature, but flag leaves continued expanding for ∼25°C d under elevated CO_2_, resulting in leaves 4 cm longer (**Figures 3A,B**). Flag leaves under high temperature expanded at the same rate regardless of CO_2_ concentration, but duration of the expansion was longer under elevated CO_2_ than ambient CO_2_ (27°C d). Therefore, flag leaves under high temperature were on average 5 cm longer under elevated CO_2_ than under ambient CO_2_.

The extension of the peduncle after ear emergence was affected by the interaction of thermal time × temperature × CO_2_ (*P* < 0.001). In both lines, the rate of peduncle extension was faster under high temperature regardless of CO_2_ concentration, but under ambient CO_2_, the duration of peduncle extension was shorter (∼10°C d). Therefore, the mainstem peduncle ambient CO_2_ + high temperature was on average 4.3 cm shorter than under elevated CO_2_ + high temperature (**Figures 3C,D**).

### Grain Growth

**FIGURE 4:**
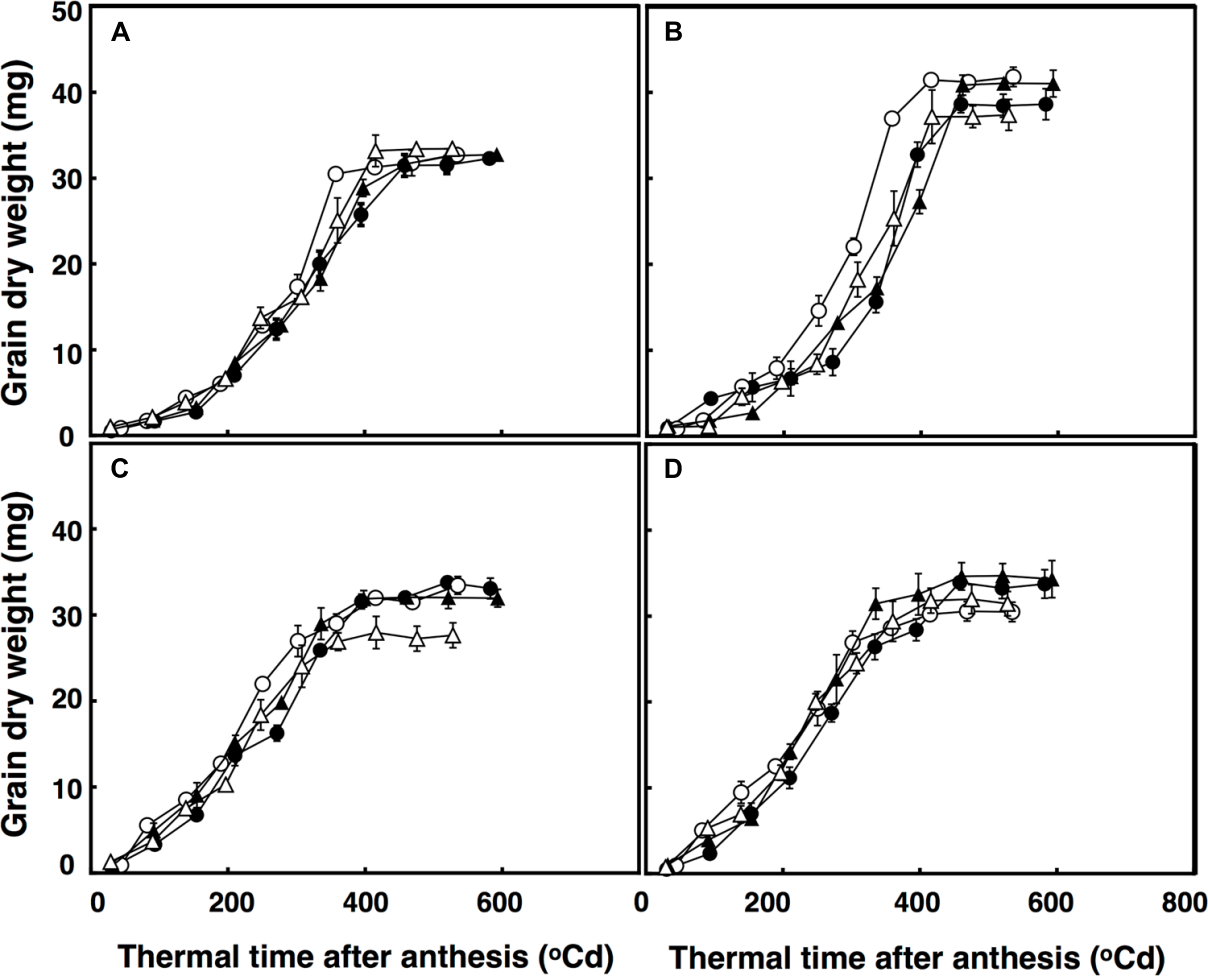
**Grain dry weight in free-tillering **(A,C)** and restricted-tillering **(B,D)** lines under well-watered **(A,B)** and terminal drought **(C,D)** conditions**. Black triangles = ACO_2_ + HT; white triangles = ACO_2_ + AT; black circles = ECO_2_ + HT; and white circles = ECO_2_ + AT. Each point is mean ± SEM of five replicates.

The lag phase of grain growth was 12% longer in the restricted-tillering line than the free-tillering line. Moreover, terminal drought reduced the lag phase by 11% on average between the two lines. The period of total grain growth was also reduced by terminal drought in both lines (by 7%). There was a significant interaction of CO_2_ × sister line × watering treatment (*P* = 0.003) for grain dry weight. The rapid growth stage in the restricted-tillering line had 17% higher rates of grain filling than the free-tillering line under both CO_2_ concentrations when well-watered (**Figure 4**). Under terminal drought, the free-tillering line had similar rates of grain filling regardless of CO_2_ concentration, while the restricted-tillering line had 12% higher rates of grain filling under elevated CO_2_. There was a second significant interaction of CO_2_ × temperature (*P* < 0.001). In this interaction, grain filling rate was 15% slower and individual mean grain dry weight was on average 7% less under ambient CO_2_ + ambient temperature than across the other treatments.

### Aboveground Biomass, Grain Yield and Yield Components

The interaction of CO_2_ × temperature was significant for total aboveground biomass (*P* = 0.004) because under elevated CO_2_ + high temperature aboveground biomass increased in both lines by an average of 18%, but not under ambient CO_2_. Terminal drought reduced aboveground biomass by 13% (*P* = 0.008), but there was no difference between the two lines (*P* = 0.704; **Table 1**). The interaction of CO_2_ × temperature (*P* = 0.002) affected HI (**Table 1**) which increased from 0.48 to 0.53 when crops were grown under high temperature and decreased from 0.51 to 0.46 when grown under elevated CO_2_. There were no significant main effects on HI (*P* = 0.058–0.880).

**Table 1:** Sources of variation for aboveground biomass (g m^-2^), grain yield (g m^-2^) and harvest index (HI) in a free-tillering and restricted-tillering line grown under ECO_2_ (elevated CO_2_) and ACO_2_ (ambient CO_2_), HT (+3°C above ambient temperature) and AT (ambient temperature), and well-watered and terminal drought conditions.

Source of variation	Aboveground biomass (g m^-2^)			Grain yield (g m^-2^)	HI		
ACO_2_	1313.0			507.0	0.51		
ECO_2_	1791.0			659.0	0.48		
AT	1524.0			578.0	0.50		
HT	1580.0			588.0	0.49		
Free tillering	1568.0			598.0	0.51		
Restricted tillering	1537.0			568.0	0.48		
Well-watered	1664.0			642.0	0.50		
Terminal drought	1441.0			524.0	0.49		

LSD	163.5			59.0	0.03		

		AT	HT			AT	HT
Interactions	ACO_2_	1407.0	1220.0	ns	ACO_2_	0.48	0.53
	ECO_2_	1642.0	1941.0		ECO_2_	0.51	0.46

LSD		231.3				0.04	

*Means of main or interaction terms and LSD (least significant difference) value at 0.05 significance level. The notation “ns” stands for not significant*.

Grain yield was not affected by any interaction of CO_2_, temperature or terminal drought for both lines (*P* = 0.105–0.996; **Table 1**). Furthermore, there was no difference in grain yield between lines (*P* = 0.309), or between temperatures (*P* = 0.739). However, main effects of elevated CO_2_ and terminal drought affected both lines (*P* < 0.001 for both) in opposite directions. While elevated CO_2_ increased grain yield on average by 30%, terminal drought reduced it by an average of 19% (**Table 1**). Ear number m^-2^ in both lines contrasting in tillering was not affected by any interactions (*P* = 0.104–0.670). Ear number was also not affected by main effects of temperature (*P* = 0.516) or terminal drought (*P* = 0.147; **Table 2**). However, there were 12.5% more ears m^-2^ in the free-tillering line than the restricted-tillering line (*P* = 0.036) and elevated CO_2_ increased ear number m^-2^ in both lines by an average of 18% (*P* = 0.004).

**Table 2:** Sources of variation of ears per m^-2^, grains per ear and single grain weight (mg) ina free-tillering (FT) and a restricted-tillering (RT) line grown under ECO_2_ (elevated CO_2_) and ACO_2_ (ambient CO_2_), HT (+3°C above ambient temperature) and AT (ambient temperature), and well-watered and terminal drought conditions.

Source of variation	Ears per m^2^	Grains per ear	Individual grain weight (mg)		
ACO_2_	417.9	37.0	33.7		
ECO_2_	492.2	39.4	34.5		
AT	446.9	39.1	33.5		
HT	463.2	37.3	34.7		
Free tillering	481.9	38.7	32.2		
Restricted tillering	428.2	37.7	36.1		
Well-watered	473.4	38.7	36.3		
Terminal drought	436.7	37.7	32.0		

LSD	50.1	2.0	1.9		

				FT	RT
Interactions	ns	ns	WW	32.8	39.7
			TD	31.5	32.4

LSD				2.6	

*Means of main or interaction terms and LSD (least significant difference) value at 0.05 significance level. The notation “ns” stands for not significant*.

Grain number per ear in both lines was not affected by any interactions (*P* = 0.077–0.924). Both lines had on average 38.2 grains per ear under ambient and well-watered conditions (*P* = 0.300) and they were not affected by temperature (*P* = 0.074) or terminal drought (*P* = 0.347; **Table 2**). Elevated CO_2_ was the only significant main effect (*P* = 0.021), which increased grain number per ear by 6.5% compared with ambient CO_2_.

Individual grain dry weights were affected by the interaction of sister line × watering regime after anthesis (*P* = 0.002) with 21.5% heavier grains in the restricted-tillering than the free-tillering line under well-watered conditions, but not under terminal drought (**Figure 4**). There were no CO_2_ (*P* = 0.390) or temperature effects (*P* = 0.217) on individual grain dry weight.

### Leaf Net Photosynthesis and Transpiration Rates, Instantaneous Transpiration Efficiency (TE_i_) and Leaf Water Potential (Ψ_Leaf_)

**FIGURE 5:**
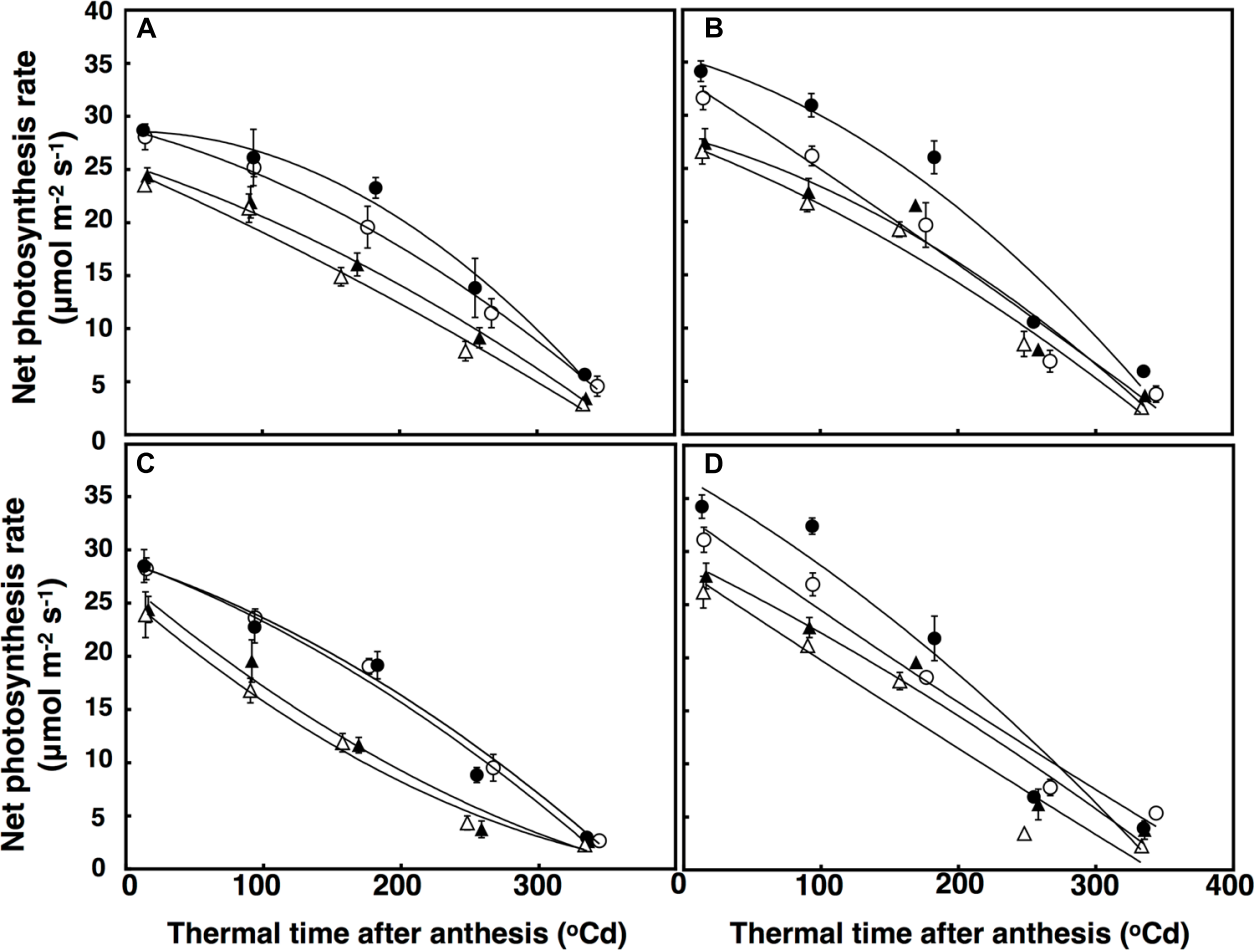
**Leaf photosynthesis rate (μmol m^-2^ s^-1^) under well-watered **(A,B)** and terminal drought conditions **(C,D)** in the free-tillering and restricted-tillering lines**. Black triangles = ACO_2_ + HT; white triangles = ACO_2_ + AT; black circles = ECO_2_ + HT; and white circles = ECO_2_ + AT. Each point is mean ± SEM of five replicates.

Leaf net photosynthetic rate was fitted using a polynomial regression including g quadratic terms, which accounted for 86% of the variation (**Figure 5**). Leaf net photosynthesis rate after anthesis was affected by a significant interactive effect of thermal time × CO_2_ × sister line (*P* = 0.041). In this interaction, the restricted-tillering line had net photosynthesis rates 16% higher on average than the free-tillering line. Under elevated CO_2_, leaf net photosynthesis rates were higher in the restricted-tillering line (25%) than in the free-tillering line (14%) and the decline in leaf photosynthetic rate with thermal time after anthesis was faster in the restricted-tillering line and slower in the free-tillering line (**Figure 5**). On average, the rates of leaf photosynthesis were 10% higher in the restricted-tillering line than in the free-tillering line across all treatments (*P* < 0.001; **Figure 5**). Under terminal drought, the net photosynthesis rate declined almost linearly, whereas the well-watered treatment reduced this decline (*P* < 0.001).

**FIGURE 6:**
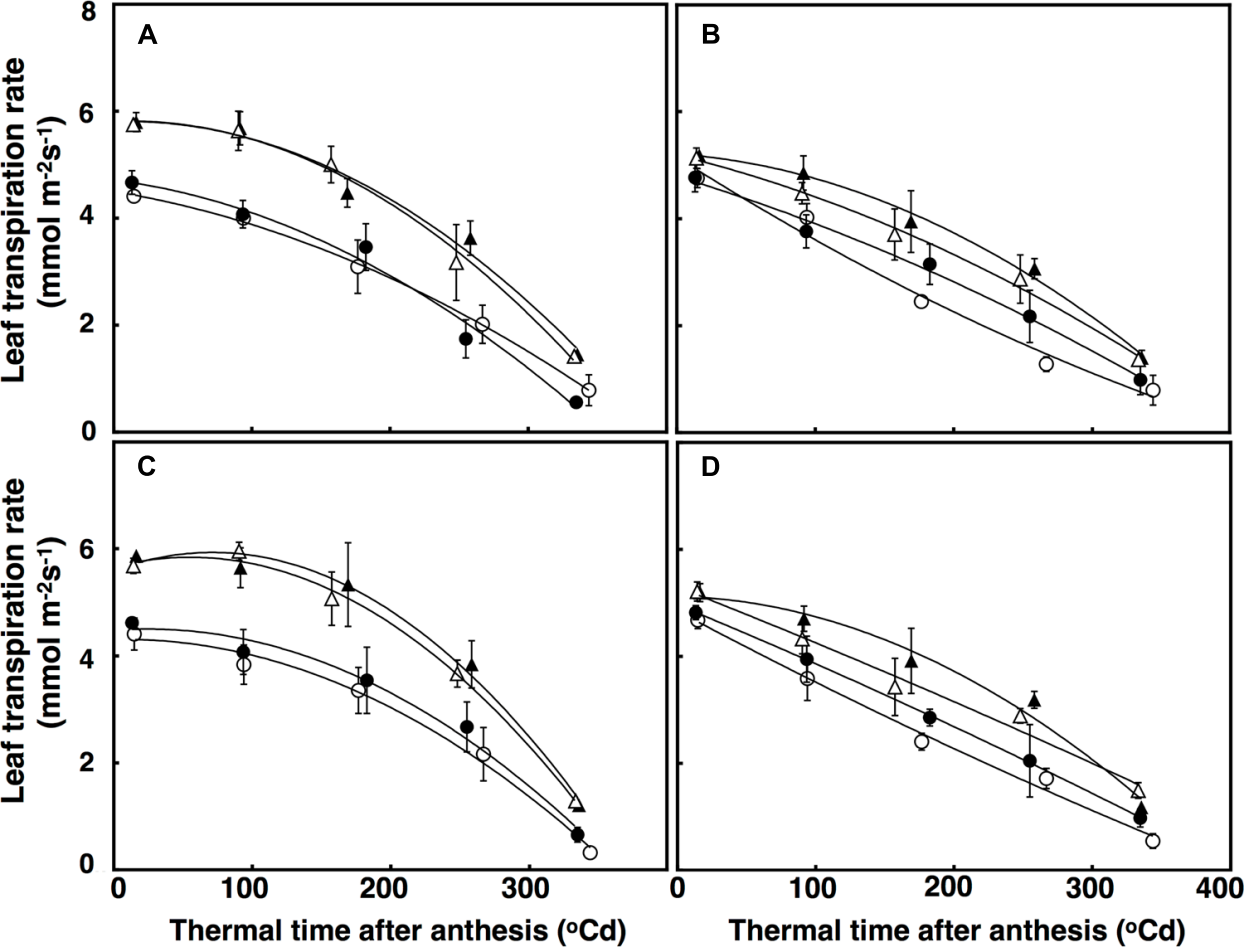
**Leaf transpiration rates (mmol m^-2^ s^-1^) under well-watered **(A,B)** and terminal drought conditions **(C,D)** in the free-tillering and restricted-tillering lines**. Black triangles = ACO_2_ + HT; white triangles = ACO_2_ + AT; black circles = ECO_2_ + HT; and white circles = ECO_2_ + AT. Each point is mean ± SEM of five replicates.

Leaf transpiration rate over time was fitted using a polynomial regression with a quadratic component, which accounted for 72% of the variation (**Figure 6**). There was a significant interaction of CO_2_ × sister line for leaf transpiration rate (*P* = 0.005) with leaf transpiration rates decreasing more under elevated CO_2_ in the free-tillering line (32%) than in the restricted-tillering line (22%; **Figure 6**). Leaf transpiration rates at anthesis in both sister lines increased by an average of 35% across all combinations (*P* < 0.001) when crops were grown under high temperature. The difference in transpiration rate between ambient and high temperature was maintained after anthesis. Leaf transpiration rate in the restricted-tillering line declined almost linearly after anthesis and was on average 12% lower than the free-tillering line, which declined non-linearly after anthesis. Terminal drought did not affect leaf transpiration rates (*P* = 0.734).

**FIGURE 7:**
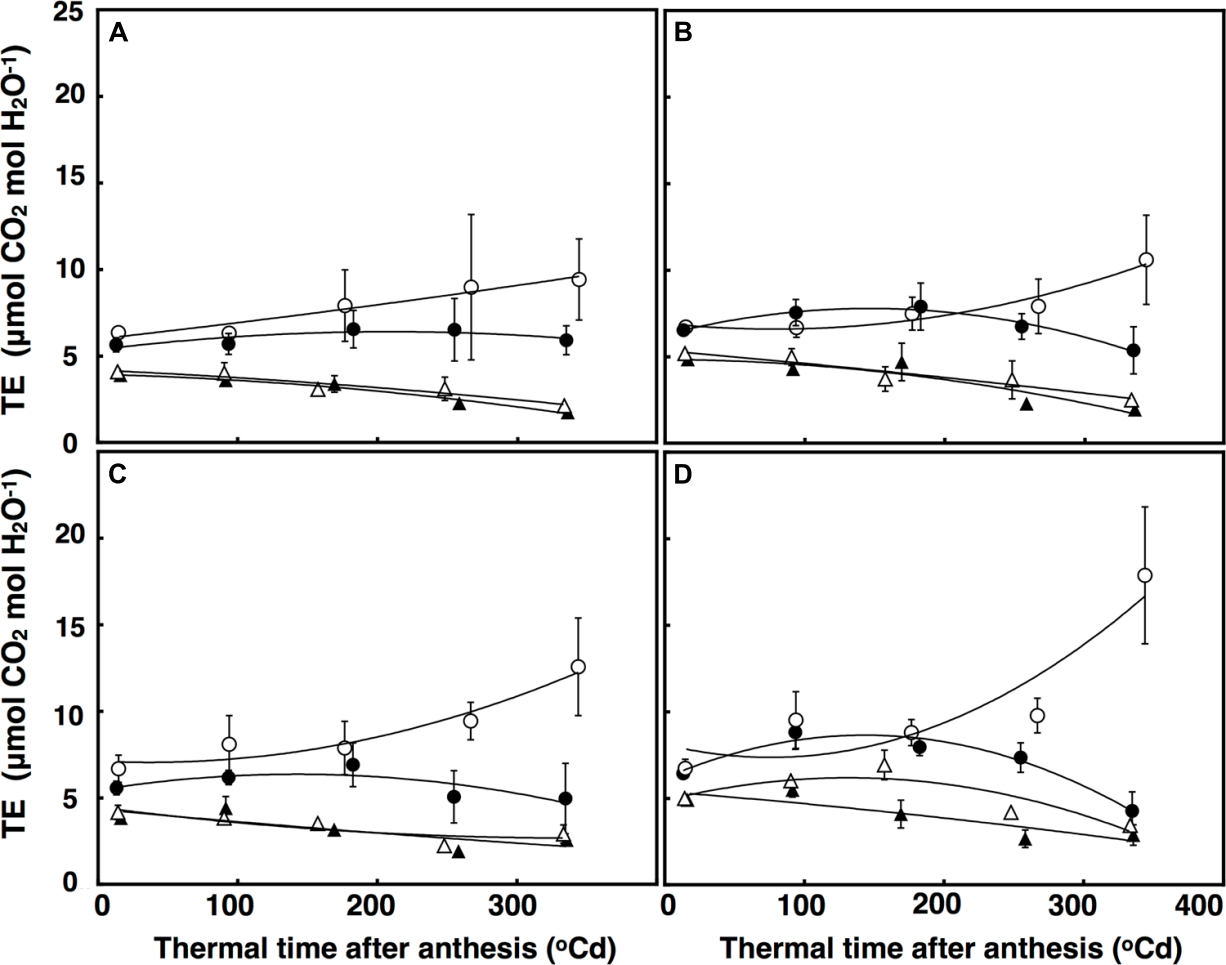
**Transpiration Efficiency (μmol CO_2_ mol H_2_O^-1^) under well-watered **(A,B)** and terminal drought conditions **(C,D)** in the free-tillering and restricted-tillering lines**. Black triangles = ACO_2_ + HT; white triangles = ACO_2_ + AT; black circles = ECO_2_ + HT; and white circles = ECO_2_ + AT. Each point is mean ± SEM of five replicates.

Transpiration efficiency over time was fitted using a polynomial regression, which explained 84% of the variation (**Figure 7**). There was a significant interaction of thermal time × CO_2_ × temperature (*P* = 0.048) for TEi. Elevated CO_2_ and ambient temperature increased TEi in both lines after approximately 320°C d, but at high temperature, TEi decreased in the restricted-tillering line under terminal drought. Terminal drought increased TEi in both lines by an average of 12% (*P* = 0.043). TEi in the restricted-tillering line was on average 20% higher than in the free-tillering line (*P* = 0.003; **Figure 7**).

## Discussion

Elevated CO_2_ increased grain yield in both lines contrasting in tillering by increasing grain number per ear. Under elevated CO_2_ there were more florets at anthesis than under ambient CO_2_. The potential number of florets did not increase, but floret mortality decreased. This finding supports the first hypothesis that elevated CO_2_ increases grain number per ear by reducing the rate of floret mortality. The carbon resources needed to produce florets are insignificant compared to that needed to support floret growth and development (Kirby, 1988; Ferrante et al., 2013a). Therefore, the high rates of net leaf photosynthesis under elevated CO_2_ most likely provided sufficient carbon assimilates (Balaguer et al., 1995; Palta and Ludwig, 2000) that enabled greater survival of competent florets. Greater availability of carbon assimilates have previously been suggested as the cause of floret mortality reduction (Siddique et al., 1989; Miralles et al., 1998). The greater dry weight of the ear and longer peduncle and flag leaf under elevated CO_2_ also indicate that the high rate of net leaf photosynthesis under elevated CO_2_ may have increased the availability of carbon assimilates. Despite increasing fertile florets in the main stem by 42%, elevated CO_2_ only increased total number of grains per ear by around 6.5%. This may be due to intraplant competition for assimilates. Main stem develops earlier than the other tillers, and supposedly, more grains to fill in the main stem could reduce carbon assimilates at for the tillers negating the gain in fertile florets. Another possibility would be that more fertile florets in the tillers are reducing the development grains on basal or apical spikelets.

The number of floret deaths at anthesis was hastened under the combination ambient CO_2_ + high temperature, reducing the potential number of grains per ear at anthesis in both lines. However, under the combination elevated CO_2_ + high temperature the rate of floret death was not affected, indicating that the hastening effect of high temperature was mitigated by elevated CO_2_ and hence the potential grain number per ear was not affected. Time to maturity was hastened under the combination of elevated CO_2_ + high temperature because elevated CO_2_ did not mitigate the effect that high temperature had on phenology. This may be due to better efficiency of photosystem II, as found in other C3 plants (Martínez-Carrasco et al., 2005; Aranjuelo et al., 2008), which reduces photorespiration (Bunce, 1998; Long and Bernacchi, 2003; Long et al., 2006; Sage and Mckown, 2006; Ainsworth et al., 2012) and can improve accumulation of assimilates for floret survival.

There was no difference in grain yield between the two sister lines contrasting in tillering under any treatment, despite the presence of more tillers in the free-tillering line under elevated CO_2_. Although tillering in the restricted-tillering line was not affected by elevated CO_2_ or high temperature, leaf extension, and leaf area do respond to elevations in CO_2_ (Dias de Oliveira et al., 2015) as well as leaf photosynthetic rates. Therefore, total canopy photosynthesis was probably similar between both lines. More tillers in the free-tillering line resulted in more grains per unit area, but the average individual grain dry weight was lower than the restricted-tillering line. The restricted-tillering line had fewer grains per unit area than the free-tillering line, but individual grain dry weight was higher. Potential grain size, which is correlated with grain dry weight, is determined by the size of the carpels within the florets (Siddique et al., 1989; Calderini et al., 2001), and presumably grain number was adjusted according to the potential grain size (Egli, 2006; Sadras and Egli, 2008). Although both lines had similar grain numbers per ear, it cannot be assumed that the potential grain size was the same. It is well understood how environmental variation affects final grain size, but not how they affect potential grain size (Slafer et al., 1996). It is likely that the changes in atmospheric CO_2_ concentration, temperature and water regime studied here did not affect potential grain size, since the plasticity in grain size has a narrow range when compared to grain number (Sadras, 2007). It is possible that final grain size was influenced by the rates of net leaf photosynthesis, which was higher in the restricted-tillering line than the free-tillering line. This could be an indication of how the sink influences the carbon source (Aranjuelo et al., 2011), but also that more assimilates were available per grain in the restricted-tillering line. Furthermore, it is possible that increased grain number per unit area reduces mean grain size (Loss et al., 1989; Siddique et al., 1989; Acreche and Slafer, 2006).

The greater number of ears m^-2^, grains per ear, grain size and leaf photosynthetic rates under elevated CO_2_ reduced the negative impact that terminal drought had on grain yield when crops were grown under ambient CO_2_ and terminal drought conditions in both sister lines. However, terminal drought reduced grain dry weight in both sister lines in this study, regardless of CO_2_ concentration or temperature. Terminal drought was induced by withdrawing irrigation when 50% of plants reached anthesis and it is, therefore, likely that the water deficit immediately after anthesis reduced grain dry weight through reduced cell expansion (Borrás et al., 2004). Furthermore, terminal drought reduced assimilation of carbon for grain filling in both lines (Pheloung and Siddique, 1991; Kobata et al., 1992; Palta et al., 1994) affecting grain growth rates.

Grain growth had a longer lag phase (period of slow grain filling) in the restricted-tillering line, and in both sister lines the lag phase was longer under well-watered conditions. The difference in final grain dry weight between the two isogenic lines for tillering under well-watered conditions resulted mainly from the faster rates of grain filling in the restricted-tillering line compared with the free-tillering line. This was probably a consequence of fewer grains per m^2^ and larger potential grain size, supported by higher carbon assimilation rates maintained after anthesis. Under terminal drought, the duration of grain filling decreased in both sister lines. Therefore, the plateau phase (maximum grain dry weight) was reached earlier, hence the reduction in grain size under terminal drought.

In both lines contrasting in tillering, elevated CO_2_ increased TEi, more as a result of reduced rates of transpiration in the free-tillering line, and increased photosynthetic rates in the restricted-tillering line. Leaf photosynthetic rates in both lines decreased at different rates as leaf senescence progressed under elevated CO_2_. Hastened phenology or limited genetic potential may have restricted biomass accumulation from higher photosynthetic rates. This limitation was previously observed in the vigorous growth breeding line 38–19, in which accelerated phenology and reduced tillering limited higher biomass accumulation despite higher leaf photosynthesis rates (Dias de Oliveira et al., 2013).

## Conclusion

Any prediction of increased grain yield due to more tillers produced was not attained because the trade-off between yield components limited grain yield in the free-tillering line. The variables of climate change did not affect the number of potential florets on the mainstem or the trade-off between yield components that limited grain yield. The number of competent florets on the mainstem at anthesis increased under elevated CO_2_ presumably, because of a greater carbon source through increased leaf area and photosynthetic rate, although the final grain number was not increased proportionally. The weight of grains in the free-tillering line was not increased, limiting any potential advantage of the free-tillering line over the restricted-tillering line under elevated CO_2_, high temperature and terminal drought conditions. This study indicates that selection of wheat germplasm with a greater potential number of florets can improve grain yield under future climates, because the amount of carbon assimilates to produce florets is very small and was not affected by any of the climate change variables. Greater photosynthetic capacity under elevated CO_2_, between stem elongation and anthesis, could support floret survival resulting in more competent florets at anthesis.

## Conflict of Interest Statement

The authors declare that the research was conducted in the absence of any commercial or financial relationships that could be construed as a potential conflict of interest.
